# Time-course ATAC-seq and RNA-seq analysis of porcine synovium-derived mesenchymal stem cells under in vitro osteogenic induction

**DOI:** 10.1186/s13072-026-00668-z

**Published:** 2026-04-01

**Authors:** Shuaichen Li, Puntita Siengdee, Frieder Hadlich, Nares Trakooljul, Michael Oster, Henry Reyer, Klaus Wimmers, Siriluck Ponsuksili

**Affiliations:** 1 Research Institute for Farm Animal Biology (FBN), Wilhelm-Stahl-Allee 2, 18196 Dummerstorf, Germany; 2 Program in Applied Biological Sciences: Environmental Health, Chulabhorn Graduate Institute, Bangkok, Thailand; 3 Faculty of Agriculture, Civil and Environmental Engineering, University of Rostock, Rostock, Germany

**Keywords:** Chromatin accessibility, Mesenchymal stem cell, Osteogenic differentiation, Transcription factor, Pig breeds

## Abstract

**Background:**

Synovium derived mesenchymal stem cells (SMSCs) are considered promising for orthopedic application due to easy accessibility and strong differentiation potential. However, the transcription factors (TFs) that orchestrate the SMSCs osteogenic commitment, as well as the dynamic landscape of associated cis-regulatory elements, remain largely unclear. In addition, donor-specific epigenetic memory may lead to heterogeneous gene-regulatory profiles.

**Results:**

In this study, we isolated porcine SMSCs from two pig breeds (German Saddleback, GS; German Landrace, GL) with distinct intrinsic metabolic characteristics and profiled their dynamic chromatin accessibility and transcriptomes during osteogenic induction. GO terms related to ossification and mesenchymal cell differentiation emerged earlier in the chromatin landscape (ATAC-seq, day 7) than at the transcriptional level (RNA-seq, day 21), indicating that chromatin accessibility captures lineage-specific programs prior to overt gene expression changes. Donor-specific differences in chromatin accessibility were minimal at baseline (day 0), became evident early after induction, and diminished over time. Footprinting analysis showed stronger binding affinity of C/EBP family members in osteogenic-induced SMSCs, whereas the FOS::JUN heterodimer exhibited greater occupancy in control cells. Interestingly, RUNX2 footprints displayed a slight decrease from day 0 to day 21 despite its established role in osteogenesis. De novo motif analysis further revealed TF-binding motif in differentially accessible regions, with RUNX2/RUNX motifs enriched in regions of reduced accessibility and CEBPs enriched in regions of increased accessibility.

**Conclusions:**

This study characterizes chromatin accessibility dynamics in SMSCs during osteogenic differentiation, driven mainly by differentiation state and time rather than donor metabolic differences. Integrated ATAC-seq/RNA-seq highlights key transcription factors and networks guiding osteogenic commitment, supporting porcine SMSCs as a translational model for bone regeneration.

**Supplementary Information:**

The online version contains supplementary material available at 10.1186/s13072-026-00668-z.

## Background

Over the past two decades, Mesenchymal Stem Cells (MSCs) have attracted growing interest in preclinical research due to their multi-lineage differentiation potential and broad availability from nearly all mesodermal-derived tissues, such as bone marrow, adipose tissue, umbilical cord, and synovium. Although MSCs from various tissues share common features, they also possess distinct properties based on their origin [1]. Among these, Synovium-derived MSCs (SMSCs) are a particularly promising candidate for the treatment of bone and joint diseases, owing to their anatomical proximity to cartilage, robust proliferative capacity, low immunogenicity, and strong innate chondrogenic differentiation potential [2]. A growing number of studies are focusing on how MSCs interact with host microenvironment via their paracrine effects to achieve therapeutic tissue repair and regeneration [3]. Nevertheless, evidence still indicates that MSCs can contribute to tissue reconstruction after transplantation through their progenitor cell properties [4]. Therefore, elucidating the molecular mechanisms governing SMSCs differentiation is essential for optimizing their therapeutic use and advancing their clinical application in regenerative medicine.

In addition to the notable potential for cartilage repair, SMSCs also show great in vitro osteogenic ability and accelerate osteoinduction in osteochondral defects [5–7]. Our group’s previous work focused the characterization of porcine SMSCs [8], detailing the gene expression patterns during in vitro osteogenic induction [9], and analyzing the accompanying shifts in the epigenetic mechanism of DNA methylation [10]. These investigations mapped several important osteogenic-related pathways, showed a significant reduction in DNA methyltransferases expression upon induction, and established a clear pig breed-dependent variation in global methylation. However, we also observed distinct expression patterns for core transcription factors (TFs), with RUNX2, a regulator of osteoblast differentiation, showing unchanged expression in our studies contrasting with previous reports [9, 11, 12].

Chromatin accessibility refer to the extent to which nuclear macromolecules can physically contact chromatinized DNA, and is subject to the occupancy and topological organization of nucleosomes, along with other chromatin-binding factors that occlude access to DNA [13]. The assay for transposase-accessible chromatin using sequencing (ATAC-seq) is a simple, rapid, and robust method to determine the sites of accessible chromatin, which can explain the mechanism behind how gene expression is regulated [14]. Recent studies described the dynamic chromatin accessibility landscapes in human and rodent MSCs during in vitro osteogenic induction [15, 16], which identified key transcriptionally active genomic regions and their regulatory elements on a genome-wide scale; however, little is known in porcine MSCs. Porcine SMSCs offer a valuable model for studying osteogenic differentiation due to their physiological and genomic similarities to humans, which might enable translational insights relevant to bone regeneration. To gain a comprehensive understanding of the gene regulatory mechanism controlling osteogenesis in porcine SMSCs, it is essential to examine the dynamic changes in chromatin accessibility.

Building on our previous work suggesting pig-breed-associated epigenetic variation, we profiled chromatin accessibility (ATAC-seq) and gene expression (RNA-seq) across an osteogenic induction time course with matched uninduced controls in porcine SMSCs from German Saddleback (GS) and German Landrace (GL). Our primary goal was to define the transcriptional and chromatin accessibility dynamics underlying osteogenic differentiation. In parallel, we assessed whether these dynamics differed by breed; however, the dominant patterns were largely conserved between GS and GL in this dataset. We therefore reconstructed gene regulatory networks and performed footprinting analyses to prioritize transcription factors driving osteogenic transitions in the shared program, while reporting breed-associated signals where supported.

## Methods

### SMSCs culture and osteogenic differentiation

Animal care and tissue collection were approved by the Animal Care Committee of the Research Institute for Farm Animal Biology and complied with institutional guidelines and EU Directive 86/609/EEC. Samples were collected at the institute’s slaughterhouse; animals received no experimental interventions, and slaughter was performed under applicable legal and ethical standards with measures to minimize pain and discomfort.

The SMSCs at passage 1 were acquired from our cell stock. The procedures for cell isolation and culture, cell marker characterization, and trilineage differentiation were described in detail in our previous work [8], as well as the osteogenic induction [9, 10]. These SMSCs expressed stemness markers CD44, CD29, CD90, and CD105 (flow cytometry) and demonstrated tri-lineage differentiation into osteogenic lineages [8–10]. Briefly, cells were isolated from three GS pigs and three GL pigs, all of which were male and 59 days of age. After two passages, cells from the three individuals of each breed were pooled to reduce individual variation. When SMSCs at passage 3 reached 80% confluence, cells for osteogenic differentiation group (Ost) were incubated with osteogenic differentiation medium, while cells for control group (Con) remained in complete culture medium. The culture medium was half-changed every 3–4 days for each group. On culturing days (D) 0, 7, 14, and 21, cell samples from each breed and group were collected for Alizarin staining, Sirius staining, RNA-seq and ATAC-seq. The differentiation protocol was performed in two independent technical replicates. In total, 28 samples were analyzed with non-differentiated cells (Con) at 4 time points (D0, D7, D14, D21) × 2 breeds × 2 technical replicates (*n* = 16) and differentiating cells (Ost) at 3 time points (D7, D14, D21) × 2 breeds × 2 technical replicates (*n* = 12).

### Alizarin red staining and Sirius red staining

The mineralization and collagen production were determined by Alizarin red and Sirius red staining, respectively [17]. At each time point, cells were rinsed with PBS, then fixed with 10% neutral buffered formalin for 30 min. After washing with distilled water, cells were incubated with Alizarin red for 30 min or Sirius red for 1 h. Following removal of unbound dye, cells were observed under Keyence BZ-X810 microscope.

### RNA-seq sample processing and data analysis

Total RNA from cells at each time point was isolated using TRI Reagent (Sigma-Aldrich, Taufkirchen, Germany), followed by purification with the RNeasy MinElute Cleanup Kit and on-column DNase digestion using the RNase-Free DNase Set (both from Qiagen, Hilden, Germany). RNA purity and integrity were assessed using NanoDrop ND-2000 (Thermo Fisher Scientific, Dreieich, German) and Bioanalyzer 2100 (Agilent Technologies, Waldbronn, Germany). The RNA libraries were prepared using the Illumina Stranded mRNA preparation kit (Illumina, San Diego, USA) and sequenced on the Illumina NextSeq 2000 at the Research Institute for Farm Animal Biology (FBN), Germany.

Raw sequencing reads were pre-processed using Trim Galore (v0.6.10) and FastQC (v0.11.9). Clean reads were then aligned to the pig reference genome (Sscrofa 11.1, Ensembl release 114) using HISAT2 (v2.2.0). Gene-level counts were obtained using the htseq-count script from HTseq (v2.0.2) with the corresponding Sscrofa 11.1 genome annotation (Ensembl release 114). Differential expression analysis of count data was performed using DESeq2 package (v1.48.1). The model included days in culture, breed, and their interaction. Genes with padj < 0.05 and |log2FoldChange| > 1 were identified as differentially expressed genes (DEGs) for the comparison between osteogenic-induced and control SMSCs, while genes with padj < 0.05 were regarded as DEGs in the comparison between GS and GL.

### ATAC-seq sample processing and data analysis

The cell sample processing and library preparation for ATAC-seq were performed as previously described with minor modification [14, 18]. Briefly, at each time point, cells were washed with PBS, trypsinized, and assessed for viability (> 95%) before cryopreservation in aliquots of 60,000 cells in 100 µL for subsequent experiments. The Tagment DNA TDE1 Enzyme and Buffer Kit (Illumina, San Diego, USA) and MinElute PCR Purification Kit (Qiagen, Hilden, Germany) were used for transposition reaction and clean-up, respectively. After barcoding the transposed fragments, libraries were amplified by PCR and purified using AMPure XP Beads. Final libraries were multiplexed and pair-end sequenced for 2 × 109 bp at 750 pM on the Illumina NextSeq 2000 at the Research Institute for Farm Animal Biology (FBN), Germany.

We used the nf-core/atacseq pipeline (v2.1.2) in Nextflow for quality control, alignment and peak calling [19]. Narrow peaks and BAM files were imported into the DiffBind package (v3.18.0) to generate consensus peak sets and analyzed with DESeq2 package (v1.48.1) for differential peak analysis. The same statistical model used for RNA-seq data was applied. Peaks with padj < 0.05 and |log2FoldChange| > 1 were identified as differentially accessible regions (DARs) in the comparison between osteogenic-induced and control SMSCs, while peaks with padj < 0.05 were considered DARs in the comparison between GS and GL. Peak annotation was performed using the ChIPseeker package (v1.44.0), with promoter regions defined as ± 2 kb from the transcription start site (TSS).

To infer transcription factors (TFs) occupancy and changes in TF binding, we made use of TOBIAS (v0.17.1) for footprinting analysis [20, 21]. Narrow peaks and BAM files from different samples with the same state_days, regardless of breeds and replicates, were merged into a single peak set and bam file. After the correction of Tn5 transposition bias, the footprinting scores were calculated. TF motifs were retrieved from JASPAR2024_CORE_vertebrates_non-redundant database [22]. To account for transiently bound TFs that may be missed by footprinting analysis, we additionally performed sequence-based motif prediction with HOMER (v5.1) [23]. For each comparison, DARs were split into open and closed categories based on their log2FoldChange values and analyzed using findMotifsGenome.pl with default settings. Up to five enriched *de novo* motifs were selected from each run, if available.

### Time course analysis

The TCseq package (v1.32.0) was utilized to perform temporal pattern analysis of RNA-seq and ATAC-seq data [24]. Normalized gene-count and peak-count data were used to construct time course table and were grouped into nine clusters using soft clustering (Fuzzy c-means) method. Z-score-transformed values were used for visualization. Clusters showing clear, consistent up- or down-regulation trends over time were chosen based on visual inspection. To ensure robust cluster assignments, we excluded genes or peaks with membership value < 0.6 within a selected cluster prior to enrichment analysis.

### Functional enrichment analysis

Ensembl gene IDs corresponding to DEGs and DARs from each comparison were converted into NCBI gene IDs and analyzed using the clusterProfiler package (v4.16.0) for over-representation analysis [25]. For GO enrichment analysis, we focused on the biological process terms and applied the simplify function to reduce redundancy. For KEGG enrichment analysis, we primarily considered pathways related to signal transduction. Only terms with p.adjust < 0.05 and gene counts > 20 were retained. Gene and peak lists showing consistent monotonic changes from TCseq were analyzed using Reactome pathway tools with default parameters. Because no significant pathways were detected when using all TCseq-identified peaks, peaks annotated to intergenic regions were excluded prior to Reactome analysis.

### Integration analysis of RNA-seq and ATAC-seq data

We performed concordance analysis to examine the overall correlation between DEGs and DARs based on fold-change values [26]. DARs with distance to TSS > = 10 kb were excluded from this analysis. In addition, we assessed the relationship between individual promoter peaks and their nearest genes to identify cases in which promoter accessibility was significantly and positively correlated with gene expression. Pearson correlation coefficients and significance levels were calculated using the Hmisc package (v5.2-3) based on normalized gene- and peak-count data.

ANANSE (ANalysis Algorithm for Networks Specified by Enhancers) is a regulatory network method that uses enhancer-derived information to rank transcription factors likely to drive cell-state changes. Using ANANSE (v0.5.1), we reconstructed directed gene regulatory networks for osteogenic induction at days 7, 14, and 21, each compared with the matched control condition [27]. The pig genome and annotation (Sscrofa11.1) were installed from Ensembl by genomepy. For TF binding prediction, BAM files were grouped based on state_days, regardless of breed and replicates. Gene-level raw count data from RNA-seq were first converted into transcripts per million (TPM) and then imported into ANANSE network. The influence score and the full GRN were calculated by ANANSE influence. The GRNs were visualized using igraph package (v2.2.1).

## Results

**Fig. 1:**
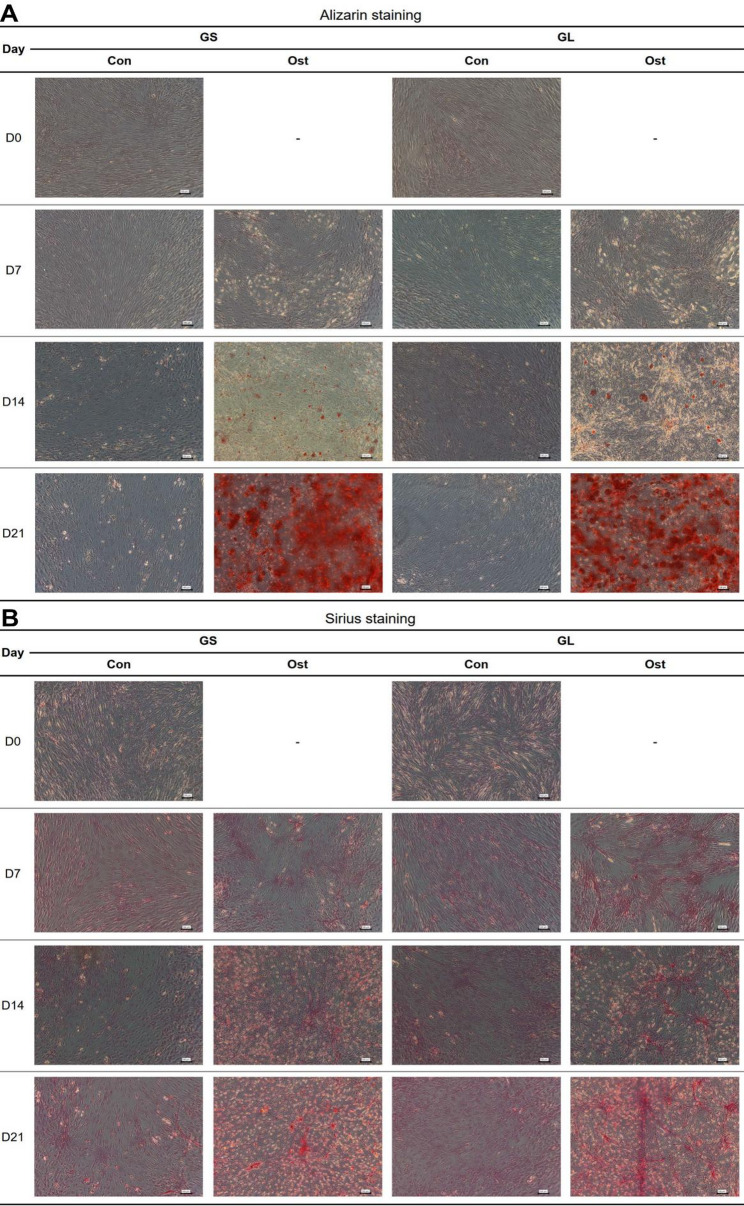
Alizarin red staining (**A**) and Sirius staining (**B**) images of control and osteogenic-induced SMSCs isolated from GS and GL pigs at day 0, 7, 14, and 21 (Scale bar: 100 μm)

Upon induction, SMSCs from GS and GL pig donors exhibited osteogenic differentiation, as indicated by calcium deposition, which appeared as black spots prior to staining and as extensive red staining following Alizarin red staining on D14 and D21 post-induction (Fig. 1A and Fig. S1). In contrast, no calcium nodules were observed in SMSCs from Control group (Con) (Fig. 1A and Fig. S1). For the Sirius red staining results, collagen deposition was first seen on D7 and progressively increased on D14 and D21 during the induction period (Fig. 1B). The SMSCs from Con group showed only a minimal amount of collagen production (Fig. 1B). The summary statistics for RNA-seq and ATAC-seq data can be found in Table S1. The 28 RNA-seq libraries yielded an average of 26.9 million reads, with an average alignment rate of 97.76%. For the ATAC-seq libraries, the average of read depth was 49.3 ± 8.6 million, yielding 82k ± 9k total peaks per sample. The Fraction of Reads in Peaks (FRiP) score averaged 0.45 ± 0.09 across all samples, indicating robust enrichment of signal within MACS2-defined peak regions. Transcription start site (TSS) enrichment averaged 5.76 ± 0.7, consistent with high data quality and a substantial proportion of peaks localizing to promoter regions.

### Dynamic transcriptional landscape of porcine SMSCs during osteogenic induction

**Fig. 2:**
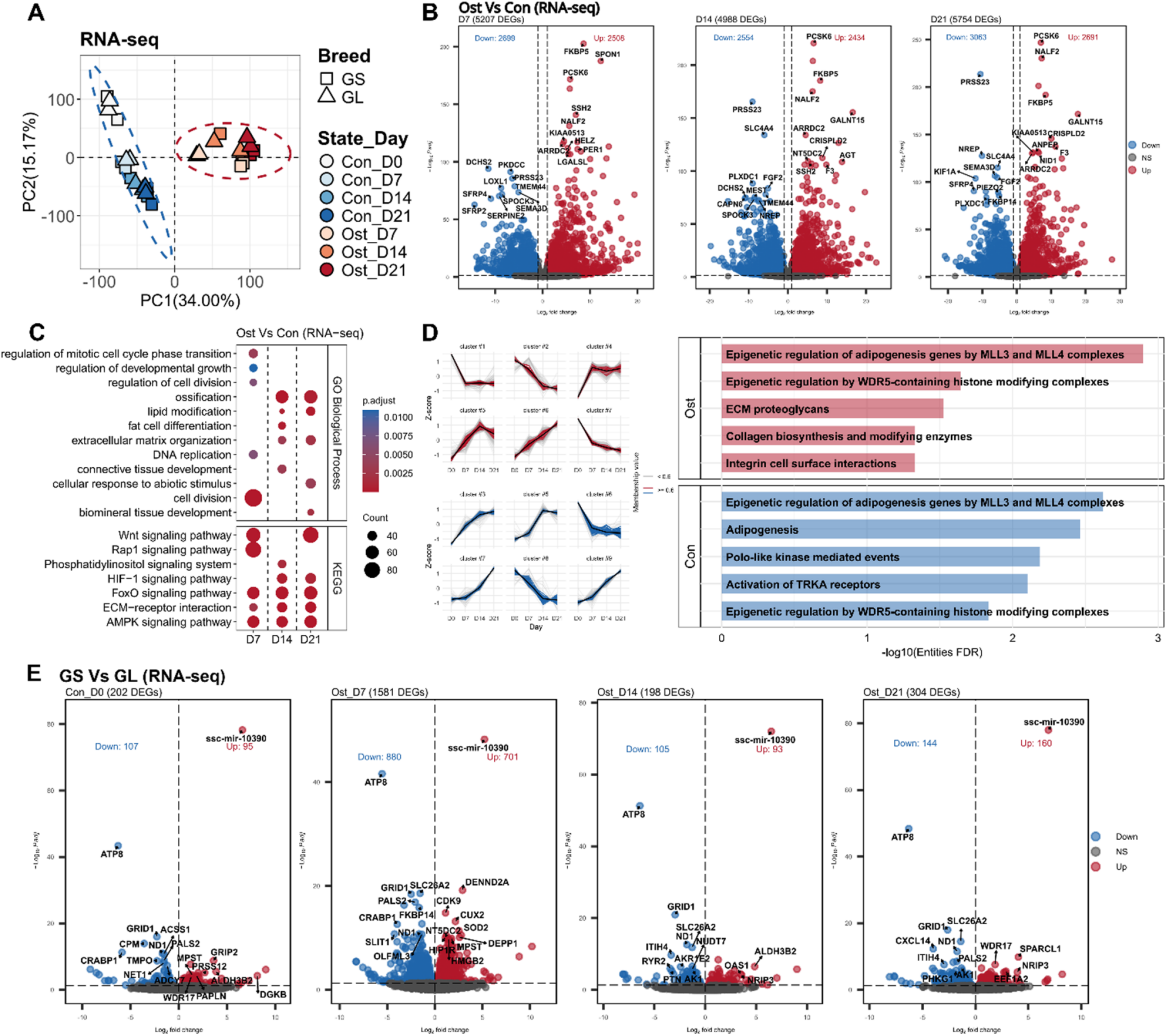
**A** Principal component analysis of RNA-seq data, with 95% confidence ellipses for the osteogenic differentiation group (Ost, red) and the control group (Con, blue). **B** Volcano plots of differentially expressed genes (DEGs) between osteogenic-induced and control Synovium derived mesenchymal stem cells (SMSCs) at each time point. Cutoff of padj < 0.05 and |log2FoldChange| > 1 was applied to defined DEGs. Up-regulated and down-regulated DEGs are colored in red and blue, respectively. The top 10 up- and down-regulated DEGs were ranked by padj and labeled using their gene symbols. **C** Biological process and KEGG pathway analysis of DEGs between osteogenic-induced and control SMSCs at each time point. **D** Reactome pathway analysis of genes showing monotonic changes over time in osteogenic timeline and control timeline, respectively. **E** Volcano plots of DEGs between GS and GL at each time point prior to and after osteogenic induction. Cutoff of padj < 0.05 was applied to defined DEGs. Up-regulated and down-regulated DEGs are colored in red and blue, respectively

The principal component analysis (PCA) result of RNA-seq data showed a clear separation between osteogenic-induced and control SMSCs along PC1, while SMSCs from different pig breeds did not form distinct clusters (Fig. 2A). We also found that SMSCs without induction followed a relatively clearer trajectory along PC2 over time, compared to osteogenic-induced SMSCs. The full list of DEGs is shown in Table S2 and S3. A total of 5,207, 4,988, and 5,754 DEGs were detected at days 7, 14, and 21, respectively, when comparing osteogenic-induced and control SMSCs (Fig. 2B). Among these genes, we observed that 4 (including *FKBP5*,* PCSK6*,* NALF2*, and *ARRDC2*) were consistently ranked among the top 10 upregulated genes in osteogenic induction cells, while *PRSS23* were consistently among the top 10 downregulated genes (Fig. 2B).

In the GO enrichment analysis of DEGs, we found cell division and cellular growth terms at day 7, such as regulation of cell division, regulation of mitotic cell cycle phase transition, and chromosome segregation (Fig. 2C, Table S4), suggesting the active cell proliferation stage in the early period of osteogenic induction. Terms engaging with ossification and biomineral tissue development were observed at day 14 and/or 21 (Fig. 2C). DEGs were also enriched in adipocyte differentiation and/or lipid modification from day 7 to 21 (Fig. 2C, Table S4), which is consistent with the notion that lineage commitment of MSCs toward adipogenesis and osteogenesis lies at opposite ends of a spectrum [28, 29]. The KEGG pathway analysis revealed the consistent participation of FoxO, ECM-receptor interaction, AMPK, and Wnt, and HIF-1 signaling pathways throughout the osteogenic induction of SMSCs (Fig. 2C, Table S4). Additionally, TGF-β and Hippo signaling pathways were found at day 7 and/or 21, together with MAPK and TNF signaling pathways from day 7 to 14 (Table S4), indicating their involvement on specific stage of induction. To identify most dynamically regulated pathways during osteogenic induction, we used temporally expressed genes for Reactome enrichment analysis. In the osteogenic-induced timeline, the top 2 pathways were related to histone methyltransferases, including *MLL3 (KMT2C)*,* MLL4 (KMT2D)*, and *WDR-5* (Fig. 2D), highlighting the dynamic contribution of epigenetic mechanisms to the osteogenic process. In addition, the marked enrichment of extracellular matrix (ECM) organization and collagen synthesis pathways demonstrated their continuous participation in the process of osteogenic differentiation (Fig. 2D). Interestingly, in the control SMSCs timeline, we found adipogenesis-related pathways (Fig. 2D), which appear to reflect the propensity for spontaneous adipogenesis with increasing time in in vitro culture.

Comparisons between samples from the control groups of GS and GL pigs revealed few DEGs, for example, 202 at day 0, 198 at day 14, and 304 at day 21; yet, a markedly higher number (1,581 DEGs) were detected at day 7 of osteogenic induction (Fig. 2E). Besides, 3 genes (*DGKB*,* KCTD19*, and ssc-mir-10390) were consistently ranked among the Top 10 upregulated DEGs in osteogenic-induced SMSCs compared to controls, as well as 3 genes (*ATP8*,* CEL*, and *TNFRSF17*) in the Top 10 downregulated DEGs (Fig. 2E, Table S3). In the KEGG enriched terms, the FoxO and Wnt signaling pathways were found at day 7 (Table S5).

### Dynamic chromatin accessibility landscape of porcine SMSCs during osteogenic induction

**Fig. 3:**
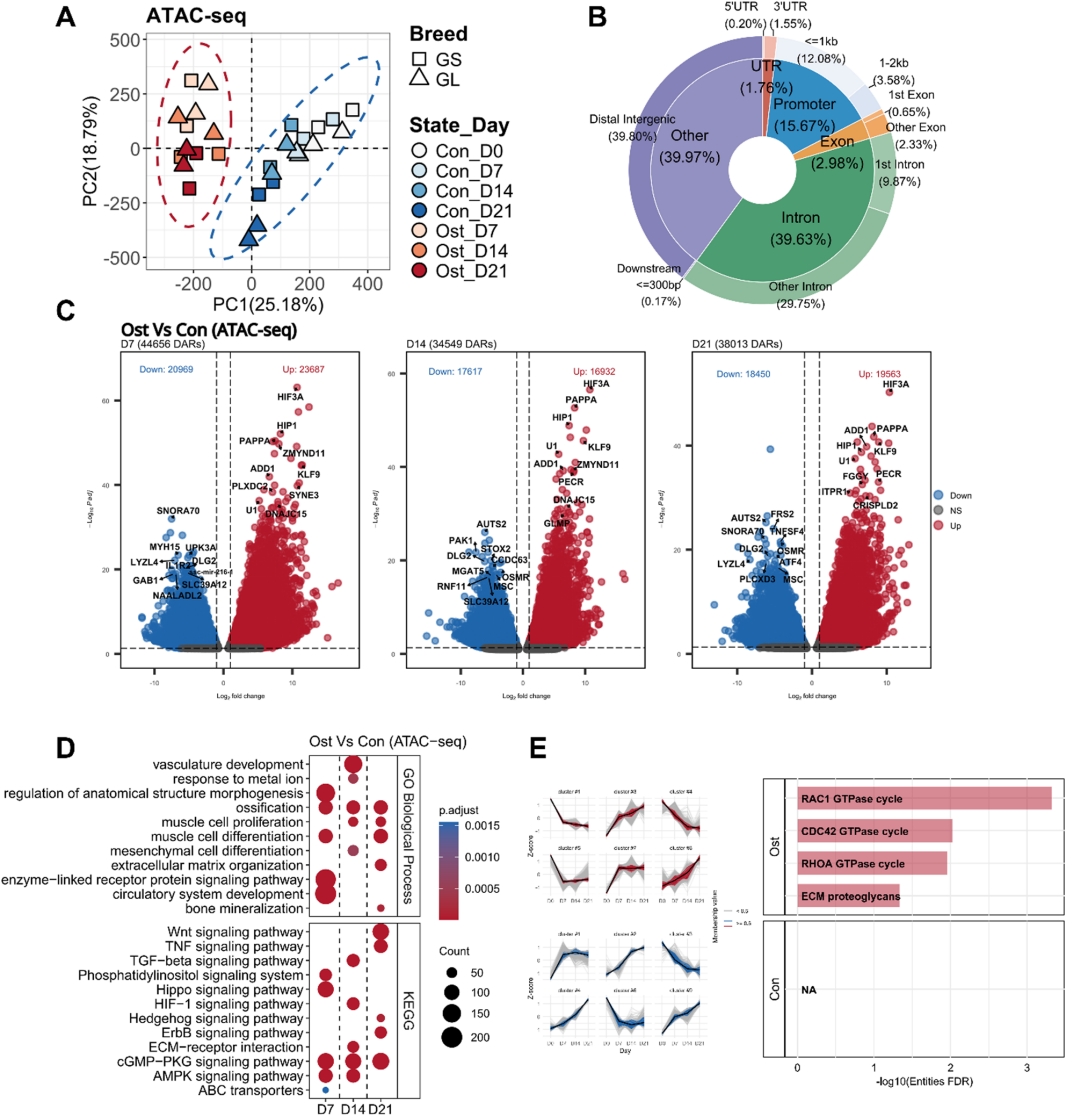
**A** Principal component analysis of ATAC-seq data, with 95% confidence ellipses for the osteogenic differentiation group (Ost, red) and control group (Con, blue). **B** Distribution of consensus peaks from all samples among different genomic regions. **C** Volcano plots of differentially accessible regions (DARs) between osteogenic-induced and control SMSCs at each time point. Cutoff of padj < 0.05 and |log2FoldChange| > 1 was applied to defined DARs. Up-regulated and down-regulated DARs are colored in red and blue, respectively. The top 10 up- and down-regulated DARs were ranked by padj and labeled using their gene symbols. **D** Biological process and KEGG pathway analysis of DARs between osteogenic-induced and control SMSCs at each time point. **E** Reactome pathway analysis of peaks showing monotonic changes over time in osteogenic timeline and control timeline, respectively

Consistent with the RNA-seq findings, the chromatin accessibility profiles allow to separate the groups in the PCA, with osteogenic-induced SMSCs being most distinct from control SMSCs (Fig. 3A). A total of 144,288 consensus peaks were detected across all samples. Among these, around 15.67% were annotated to promoter regions and other 39.97% to intergenic regions (Fig. 3B). Within the intragenic category, 1.76% of peaks mapped to UTRs, 2.98% to exons, and 39.63% to introns (Fig. 3B). Differential analysis of peaks between osteogenic and control SMSCs identified 44,656, 34,549, and 38,013 differential accessibility regions (DARs) at days 7, 14, and 21, respectively (Fig. 3C, Table S6), providing a clear overview of the global impact of in vitro osteogenic induction on chromatin accessibility landscape. Genes with persistently significant changes in chromosomal accessibility at their promoter regions during induction included *FKBP5*,* SYNE3*, and *ZFYVE21*, which showed increased accessibility, while *CPO*,* FHIT*, and *SLC4A4* displayed decreased accessibility (Table S6).

In the GO enrichment analysis of DARs, ossification term was enriched from day 7 to 21 (Fig. 3D). Other osteogenesis-related terms, including mesenchymal cell differentiation and bone mineralization, were also found at day 14 and 21, respectively (Fig. 3D). Of note, terms associated with muscle cell development were also enriched from day 7 to 21 (Fig. 3D). The results of KEGG pathway analysis highlighted the cGMP − PKG signaling, AMPK signaling, and ECM-receptor interaction pathways were fully involved in the regulation of osteogenic differentiation (Fig. 3D, Table S4). Besides, pathways, such as: Wnt, Hippo, MAPK, and Hedgehog signaling pathways were found in more than one time point (Table S4). Reactome enrichment of TCseq-identified time-course peaks highlighted ECM proteoglycan pathways, consistent with the corresponding gene-expression dynamics during induction (Fig. 3E). Moreover, several GTPase cycle pathways, containing *RAC1*,* CDC42*, and *RHOA*, were found in the osteogenic-induced timeline. No significantly enriched pathway was seen in the control timeline (Fig. 3E).

**Fig. 4:**
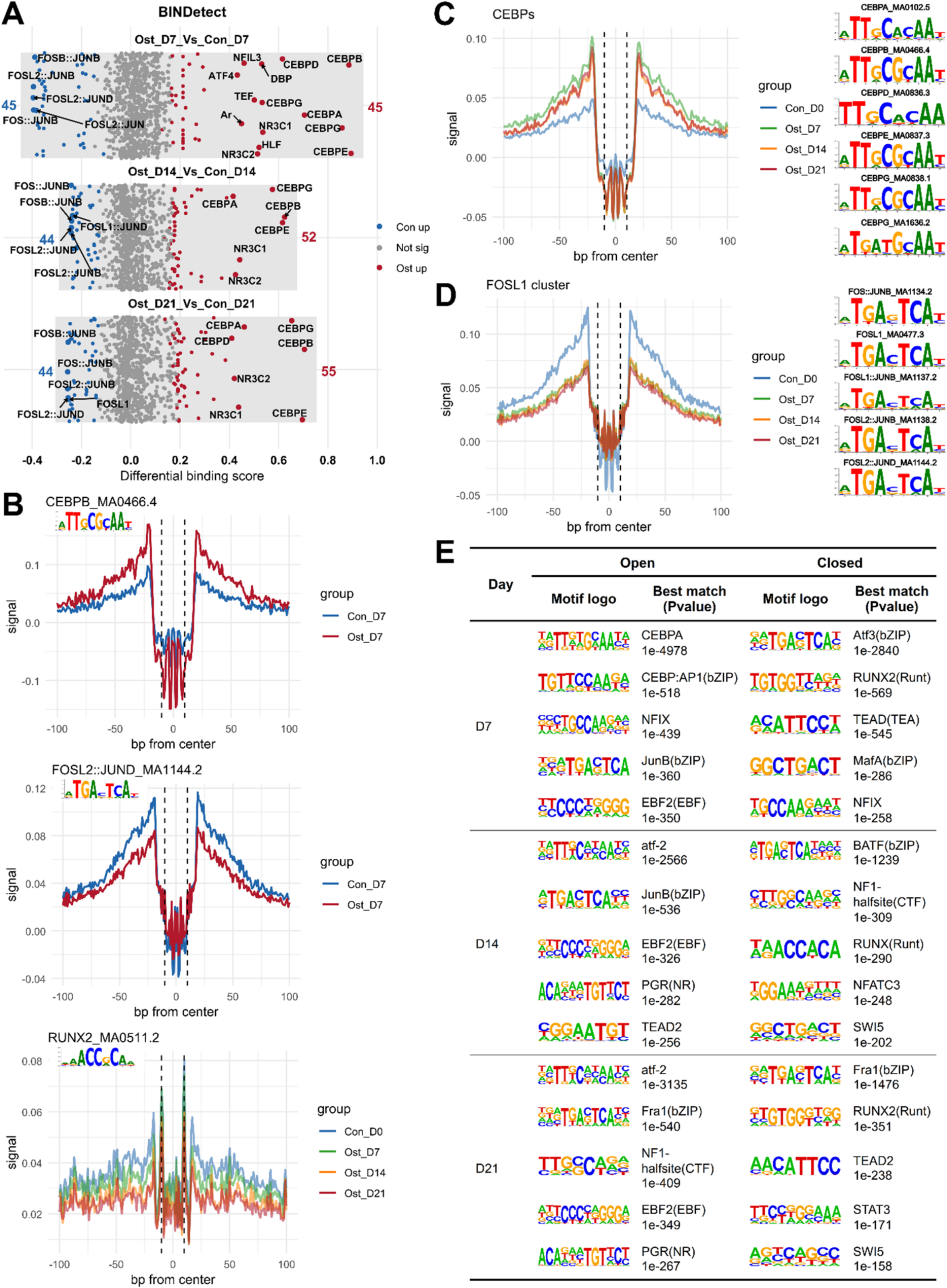
**A** Differential binding plot between osteogenic-induced and control SMSCs at each time point. TFs with differential binding scores below the 5% quantile (blue) or above the 95% quantile (red) are highlighted. **B** Visualization of footprint from individual transcription factor (CEBPB, FOSL2::JUND, and RUNX2). **C**, **D** Visualization of footprint from multiply transcription factors. Signals from TFs with similar motif logo are merged. C, CEBPs gene family (CEBPA_MA0102.5, CEBPB_MA0466.4, CEBPD_MA0836.3, CEBPE_MA0837.3, CEBPG_MA0838.1, and CEBPG_MA1636.2). **D** FOSL1 cluster (FOS::JUNB_MA1134.2, FOSL1_MA0477.3, FOSL1::JUNB_MA1137.2, FOSL2::JUNB_MA1138.2, FOSL2::JUND_MA1144.2). **E** Homer *de novo* motif enrichment analysis of open and closed DARs identified between osteogenic-induced and control SMSCs at each time point

The quantity and diversity of differential binding TFs changed very little from day 7 to 21 in the comparison of osteogenic and control cells, yet TFs with higher differential binding score tended to be observed in osteogenic differentiation (Ost) group (Fig. 4A). C/EBP gene family members (including CEBPA, CEBPB, CEBPE, and CEBPG) showed consistently elevated binding activity in osteogenic-induced cells. Conversely, the FOS::JUN heterodimeric transcription factor complex had greater genomic occupancy in the control cells (Fig. 4A). The representative peak-dip-peak patterns of *CEBPB* and FOSL2::JUND between Ost and Con cells at day 7 confirmed robust footprints (Fig. 4B). Interestingly, the footprint plot of RUNX2 from day 0 to 21 displayed a slight decrease as osteogenic induction proceeded (Fig. 4B). To better investigate the binding activities of key TFs during osteogenic induction, we combined the signals of several transcription factors that originated from same gene family or share highly similar binding motifs (Fig. 4C-D). Throughout the osteogenic timeline, we observed a significant increase in binding occupancy for the CEBP family (Fig. 4C), contrasted by a decrease for the FOSL1 cluster (Fig. 4D). Consistently, the RNA-seq data showed that the expression levels of *CEBPB* and *CEBPD* increased following osteogenic induction, while *FOSL1* expression decreased (Table S2). This result implied the increased recruitment of CEBPB and/or CEBPD to accessible chromatin to regulate and maintain the process of in vitro osteogenesis. The *de novo* motif analysis using HOMER revealed the potential binding preferences related to differentially chromatin accessibility between osteogenic-induced and control SMSCs. In line with part of the footprint findings, RUNX2/RUNX was overrepresented in DARs with lower accessibility during osteogenic induction, while CEBPA was enriched in more accessible regions at day 7 (Fig. 4E). On top of that, the components of AP-1 TF complex were found both in open and closed DARs, including, JUNB, ATF2, ATF3, Fra1 (FOSL1), MAFA, and BATF (Fig. 4E). Furthermore, we observed that EBF2 motif was consistently enriched in regions with higher accessibility in osteogenic SMSCs, yet the expression of EBF2 was decreasing, suggesting that EBF2 may not be the primary driver of the observed chromatin accessibility changes.

**Fig. 5:**
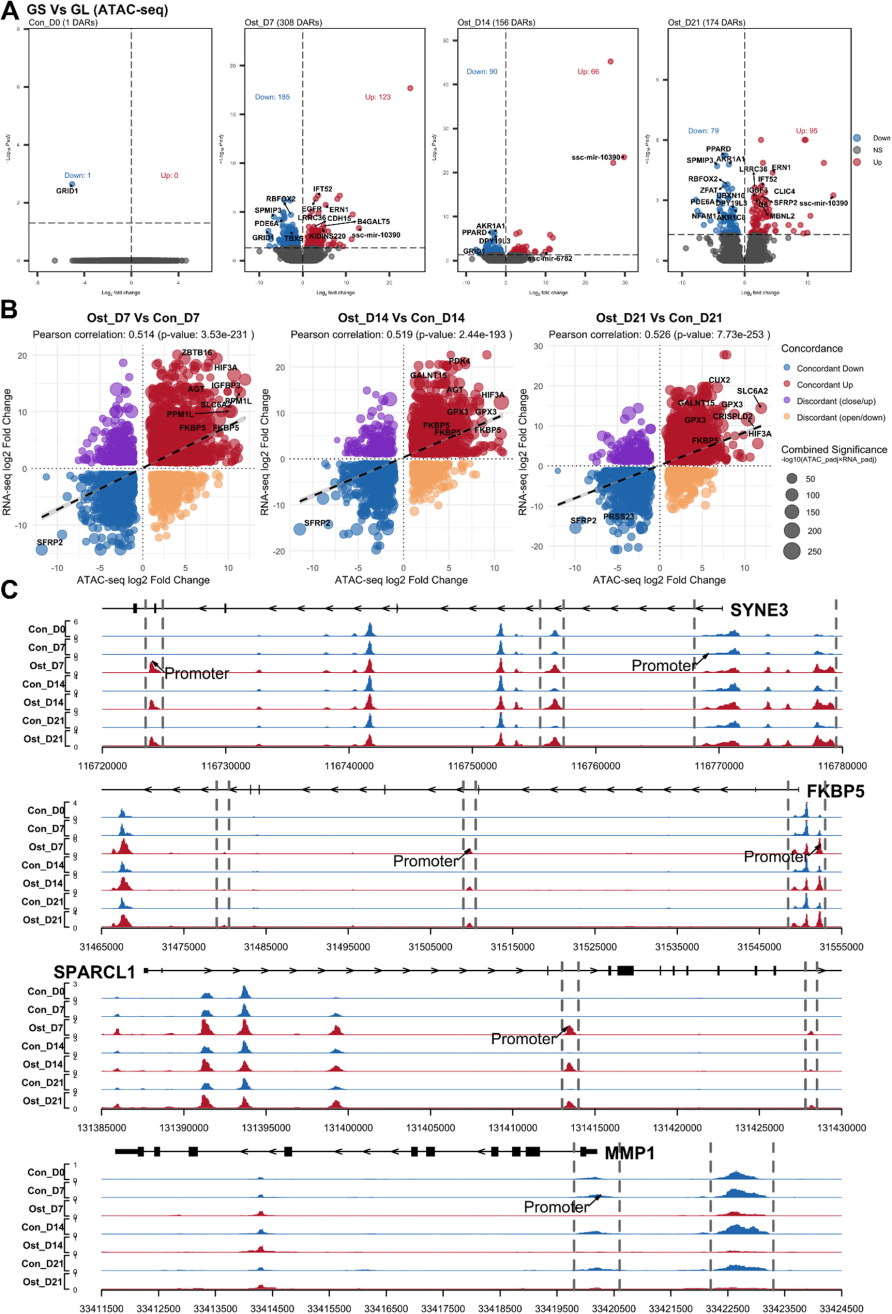
**A** Volcano plots of differentially accessible regions (DARs) between GS and GL at each time point prior to and after osteogenic induction. Cutoff of padj < 0.05 was applied to defined DARs. Up-regulated and down-regulated DARs are colored in red and blue, respectively **B** Scatter plots showing the overall relationship between DEGs and DARs from each comparison according to their log2 fold change. Concordant changes in expression and accessibility are shown in blue and red, whereas discordant changes are shown in purple and orange. **C** Genomic tracks showing differential accessibility regions between osteogenic-induced (red tracks) and control (blue tracks) SMSCs from day 0 to 21. DARs are indicated with dashed lines, and those annotated to promoter regions are explicitly labeled

For the comparisons between pig breeds, only 1 DAR was seen at day 0. After osteogenic induction, 308, 156, and 174 DARs were identified between GS and GL at days 7, 14, and 21, respectively (Fig. 5A). Among these DARs, peaks located in *ERN1*,* IL23R*,* KIDINS220*, ssc-mir-10,390, and *TMPRSS6* were always higher accessible in GS than in GL, while peaks sited in *PDE6A* and *SPMIP3* remained lower accessible during induction (Table S7). A handful of motifs were identified between breeds in the de novo motif analysis, such as, FOS, and CEBPA (Fig. S2).

### Integration of RNA-seq and ATAC-seq results

We first examined the correlation of fold changes between DEGs and DARs in each comparison. In the osteogenic-induced verse control SMSCs, we observed a significant positive correlation across all three time points, with Pearson’s r ranging from 0.514 to 0.526 (Fig. 5B). Only a couple of paired DEG–DARs were identified when comparing GS and GL from day 7 to 21, with none observed at day 0. Nevertheless, most of the detected pairs exhibited concordant changes (Fig. S3). We then investigated the correlation between each gene and its corresponding promoter peak. In total, 3841 significant gene-peak pairs (*p* < 0.05) were identified, of which 3051 showed positive correlation (Table S8). The top five genes whose expression is positively correlated with the chromatin accessibility of their promoter regions are *MEGF6*,* FKBP5*,* ALDH1L1*, *ODAPH*, and *CYRIA*. We visualized the chromatin accessibility profiles of four representative genes with differential signals between osteogenic-induced and control SMSCs during induction (Fig. 5C).

**Fig. 6:**
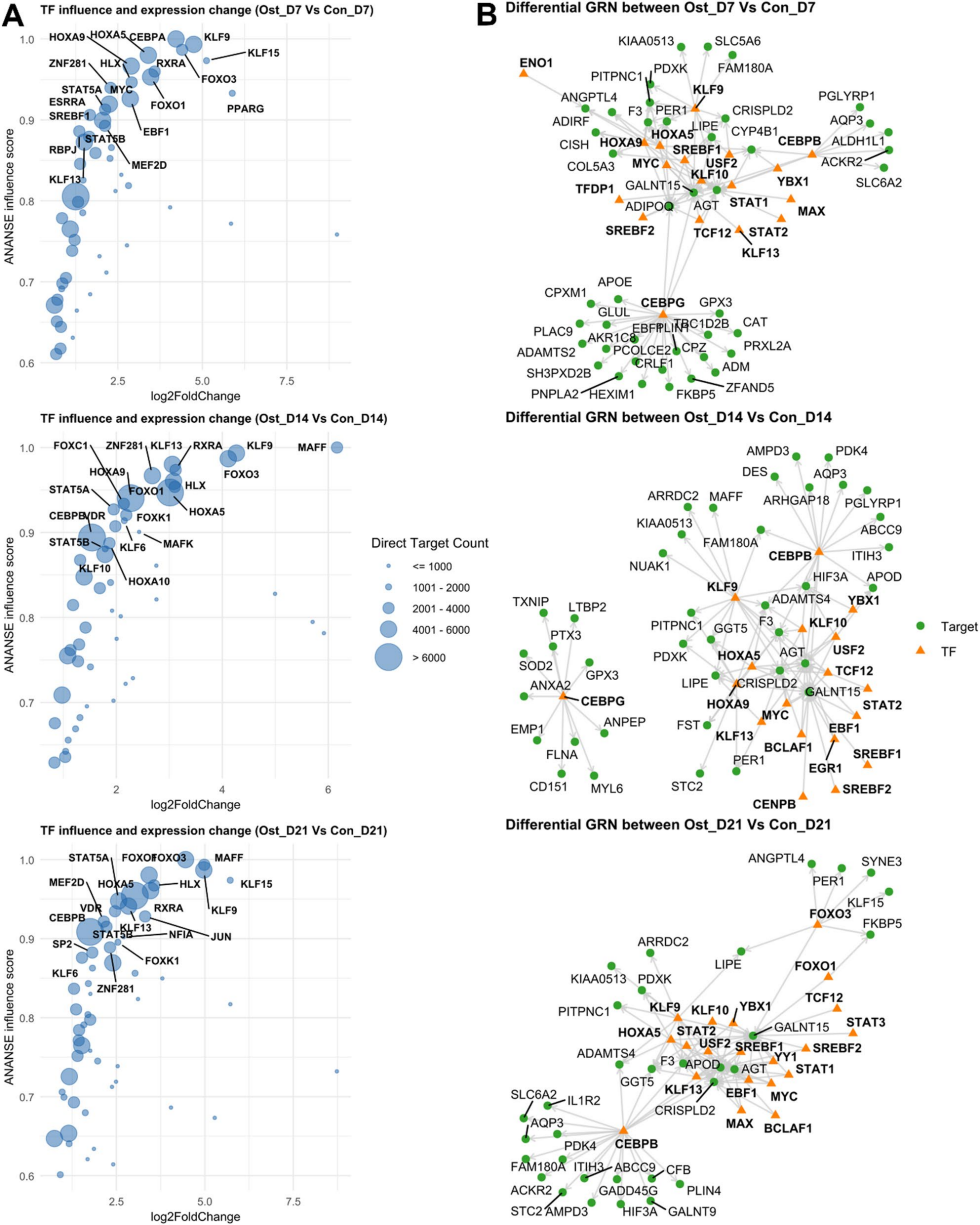
**A** Scatter plots illustrating log2FoldChange and ANANSE (ANalysis Algorithm for Networks Specified by Enhancers) influence score for transcription factors (TFs) between osteogenic-induced and control synovium derived mesenchymal stem cells (SMSCs) at each time point. The top 20 most influential TFs are labeled. The bubble size represents the number of direct target genes for that TF. **B** Differential gene regulatory network (GRNs) using the top 100 TF-target pairs, ranked by ANANSE weight values (regulatory strength), identified between osteogenic-induced and control SMSCs at each time point. The orange triangles represent transcription factors, while the green circles indicate target genes

In addition to promoters, distal regulatory elements are also essential for defining cell differentiation programs. Therefore, we employed ANANSE (ANalysis Algorithm for Networks Specified by Enhancers) to infer key TFs and gene regulatory networks (GRNs) that could lead to osteogenic differentiation. By integrating TF activity derived from ATAC-seq with RNA-seq expression profiles, ANANSE models interactions between TFs binding at distal regulatory elements and their target genes. Crucially, it functions without relying on large reference datasets, making it highly applicable to non-model organisms. Six TFs, namely KLF9, FOXO3, HOXA5, RXRA, FOXO1, and HLX, were identified as master regulators critical for osteogenic induction due to their consistently high regulatory impact (based on ANANSE influence score) across all time points (Fig. 6A). Notably, CEBPB consistently exhibited the largest number of direct target genes (Fig. 6A). Visualization of the GRNs, constructed using the Top 100 TF-target pairs for each comparison, reveals that CEBP gene family exert broad gene-regulatory effects throughout the course of osteogenic induction (Fig. 6B).

## Discussion

Osteogenic lineage commitment results from a complex interplay between transcriptional and epigenetic regulation [30, 31]. Extensive gene expression changes in porcine SMSCs during in vitro osteogenic induction were reported in our earlier study [9]. However, we did not observe a commensurate change at the methylation level during this process, which instead exhibited a clear donor-specific pattern [10]. This prompted us to explore other epigenetic alterations, such as changes in chromatin accessibility, that may occur dynamically during in vitro osteogenesis. Furthermore, previous analyses failed to identify some genes that are thought to be closely related to osteogenic differentiation in human and mouse models due to potential technical limitations and limited representation of genes on the microarrays used. Therefore, the ATAC-seq and RNA-seq data in the present study contribute to provide a comprehensive understanding of the dynamic regulation of osteogenesis in a porcine model.

This study demonstrates that highly dynamic changes in chromatin accessibility coincided with a great number of gene expression changes during in vitro osteogenic induction of porcine SMSCs. Moreover, the overall trends of these changes are mainly positively correlated. This coordinated pattern is also found to macroscopically reveal the precedence of major chromatin state shifts over transcriptional changes. For example, when comparing osteogenic-induced SMSCs and control SMSCs, the number of DEGs peaked at day 21, consistent with our earlier study, whereas the number of DARs topped out at day 7 and then slightly decreased at later stages of induction. Aligning with reports that chromatin accessibility profiles distinguish MSCs by tissue origin more robustly than transcriptome profiles [32], our data show that ossification term emerge earlier in ATAC-seq (day 7) than in RNA-seq (day 14), indicating that changes in chromatin accessibility landscape captures lineage-specific programs ahead of detectable transcriptional activation.

The results of KEGG enrichment showed the AMPK and Wnt pathways were all actively involved in the osteogenic process of porcine SMSCs. The AMPK pathway serves as a primary mechanism for maintaining cellular energy homeostasis [33], and its activation has been reported to promote bone nodule formation in vitro and inhibit adipogenesis [34, 35]. Our results implicate the dynamic regulatory role of the AMPK pathway in transcription and chromatin remodeling during osteogenesis. The crucial involvement of the Wnt pathway in skeletal development has been well established [36]. The continuous upregulation of *WNT5B* and *WNT11*, along with downregulation of Wnt inhibitors *SFRP2* and *SFRP4*, further supported the important role of this pathway in the osteogenic differentiation of porcine SMSCs. Besides, we observed significant enrichment of the MAPK signaling pathway in both the transcriptomic and chromatin accessibility landscapes, as well as the upregulation of MAPK7 at day 14 and 21, which aligns with recent functional studies confirming its role during osteogenesis of mouse MSCs [37], underscoring the key role of the MAPK pathway in osteogenic differentiation of pig MSCs. Similar with human MSCs [15], we also noted that the cGMP-PKG pathway was enriched in ATAC-seq data but not RNA-seq data during in vitro osteogenic induction, suggesting that primarily dominance at regulatory level. A recent study found that the cGMP-PKG pathway contributes to bone formation by the recovery of Wnt signaling [38]. By Reactome enrichment analysis, the ECM and collagen-related pathways were identified in the temporal-changed gene and peak clusters during osteogenic induction. The ECM is a vital non-cellular structure that not only give structural support but is invariably remodeled to regulate many biological activities [39]. The bone matrix is made up of collagen, hydroxyapatite, and a few but important non-collagenous proteins. We observed that the expression of two major non-collagenous proteins, *SPARC* (ON, Osteonectin) and *SPP1* (OPN, Osteopontin), were upregulated at different time points, as well as the easier accessible chromatin in the promoter regions of SPARC in osteogenic-induced SMSCs compared with control SMSCs, indicating their involvement at different stages of the biomineralization process. In addition, well-recognized osteogenic markers, including *BMP2* and *BMP4*, were upregulated at multiple time point during osteogenesis in both our previous work and the current study [9]. In contrast, studies on pig adipose- and bone marrow-derived MSCs have reported stable expression levels for these two genes during induction [40], potentially indicating the presence of tissue-specific differences in expression profiles.

A strong overlap of promoter-associated DARs across days 7, 14, and 21 was observed, indicating that the promoters of these genes become accessible early and remain open throughout the differentiation process. Notably, promoters of genes such as *SPARCL1*,* SYNE3*,* IQGAP2*,* PDK4*,* DUSP1*, and *FKBP5* showed increased chromatin accessibility in osteocyte-induced SMSCs, highlighting multiple key facets of osteocyte biology. In contrast, some promoter regions, including *MMP1*, displayed decreased accessibility during differentiation. We observed that *SPARCL1* was upregulated at day 14 and 21 post-osteogenic induction, while its promoter region exhibited sustained high chromatin accessibility across the entire time course. This gene is thought to be a key target of osteogenic TF regulation [41] and prior research indicates that it acts as a negative regulator of adipogenic differentiation [42]. SYNE3 contributes to the nuclear envelope and mechanosensory structures, supporting osteocytes’ role in sensing mechanical forces [43]. While IQGAP2’s function as a cytoskeletal scaffold regulating Rho GTPase signaling and cell morphology, which aligns with osteocytic dendritic process formation and mechanotransduction [44]. PDK4, a metabolic regulator, is involved in controlling energy metabolism and oxidative stress resistance during osteogenic differentiation and related calcification processes [45, 46]. Studies show that DUSP1’s modulation of MAPK pathways is pivotal in maintaining bone homeostasis and osteocyte response to mechanical and inflammatory stimuli [47]. Compared with control SMSCs, osteogenically induced cells showed increased *FKBP5* expression and greater *FKBP5* promoter accessibility, consistent with reports that FKBP5 promotes osteogenesis of human adipose-derived MSCs via the FKBP5-AKT-FOXO1 pathway or type I interferon inhibition [48, 49]. MMP1 (matrix metalloproteinase-1) is a collagenase that plays a role in remodeling the extracellular matrix and is involved in extracellular matrix degradation and contributes to cell migration, which is important in tissue remodeling and differentiation [50]. Decreased chromatin accessibility at the *MMP1* promoter during osteogenic differentiation is consistent with suppression of MMP1 expression as cells transition from proliferative MSCs to more differentiated osteocytes, reducing ECM remodeling activity [51].

Transcription factors (TFs) and their regulatory circuits are major determinants of cellular fate and cell type-specific gene expression [52]. An interesting discovery in this study is that footprint analysis revealed a stronger binding activity of C/EBP family members in osteogenic-induced SMSCs, notwithstanding the high sequence similarity among these motifs prevents us from identifying the exact TF(s) involved. It is generally believed that *CEBPA* could promote adipogenesis and inhibit osteogenesis [53, 54], but *CEBPB* and *CEBPD* seem to have more complex roles in the lineage commitment of MSCs. Some studies showed *CEBPB* and *CEBPD* are required for osteoblast mineralization [30, 55–57]; on the other hand, their pro-adipogenic activity has also been reported [58, 59]. Here we found the sustained upregulation of *CEBPB* and *CEBPD* throughout osteogenic induction, as well as a transient increase in *CEBPA* expression at day 7. The prediction of key TFs and GRNs from ANANSE also highlighted the regulatory role of C/EBP members during osteogenic induction. Given that CEBPs often require co-regulation with PPARG to drive adipogenic differentiation, and considering that *PPARG* expression was only upregulated at day 7 in our systems, we thus infer that *CEBPB* and/or *CEBPD* primarily mediate a positive regulatory role in the osteogenic differentiation of porcine SMSCs. Furthermore, this study also underscores the critical and intricate role played by AP-1 TF complex during the in vitro osteogenesis of porcine SMSCs. The AP-1 complex consists of a variety of members from the *FOS*,* JUN*,* ATF*, and *MAF* families and functions as a highly combinatorial and flexible regulatory system. It is considered that the AP-1 complex participates in the regulation of mesenchymal stem cell stemness as well as the activity of osteoblasts in bone formation [60, 61]. Contrary to changes seen in C/EBP family members, our data exhibited that the footprint occupancy of the FOS::JUN heterodimer decreased significantly upon induction. Additionally, we identified two DEGs belonging to the FOS or JUN gene family, i.e., upregulated JUN and downregulated FOSL1. It has been reported that FOSL1 is downregulated during the early phase of osteogenic induction in human MSCs, and is closely related to the regulation of other genes at later stage [62].

Although RUNX2 is recognized as a master TF in human and rodent MSCs osteogenic differentiation, this study reinforces prior transcriptomic findings [9, 40], which showed no change in *RUNX2* expression in porcine MSCs (adipose, bone marrow, or synovium), by further demonstrating no detectable change in the chromatin accessibility of the *RUNX2* promoter region during in vitro osteogenic induction. Interestingly, here we found that the stable expression of *RUNX2* is being functionally redirecting, leading to its binding at genomic regions that are becoming more compact with induction. This could suggest that in the osteogenic process of porcine MSCs, *RUNX2* is participating in a mechanism of targeted gene repression at these loci, likely mediated by post-translational modifications that alter its protein-DNA interaction preference or its association with repressive complexes. Further clarification of RUNX2’s precise role in the process of porcine bone formation will be of importance to studies involving pig regenerative medicine models.

Using the inferred stage-specific binding profiles, we examined transcription factor–target gene (TF–gene) relationships to reconstruct cell stage–specific GRNs. Networks were summarized by TF–gene interaction scores across osteogenic induction at days 7, 14, and 21. In addition to the CEBP family discussed above, *KLF9*, *FOXO3*, *RXRA*, *FOXO1*, and *MAFF* emerged as hub transcription factors in our GRNs. KLF9 and FOXO family members have documented roles in osteogenic differentiation and bone homeostasis [63–66], whereas *RXRA* as a nuclear-receptor hub acting as a central “hub” for lipid/retinoid/vitamin D signaling with context-dependent effects in osteogenic programs [67, 68]. *MAFF*, a small Maf family member, was also identified as a hub TF; related Maf factors promote osteoblast differentiation and bone formation in mice, suggesting a potential role for *MAFF* in osteogenic regulation [69].

The GS pig and GL pig breeds differ in growth performance, metabolism, and body composition [70–72]. Such metabolic profile may be reflected to some degree in the epigenetic mechanisms of in vitro osteogenesis. DNA methylation is widely regarded as a relatively stable epigenetic mark that can encode long‑term, sometimes heritable, information about cellular or donor background, including metabolic phenotype, and this matches our previous findings where GS- and GL-derived SMSCs retain breed-specific methylation differences across osteogenic or adipogenic differentiation [10, 73]. In contrast, chromatin accessibility is known to change rapidly and bidirectionally during cell fate transitions, often tracking immediate transcription factor occupancy and signaling rather than long-term history [74]. The donor-derived DNA methylation memory was retained throughout the induction process [10], in contrast to chromatin accessibility, which showed no obvious segregation based on donor source in this study. Together with our previous work, this supports the view that DNA methylation can act as a relatively stable record of donor-specific epigenetic memory, whereas chromatin accessibility is more rapidly remodelled during osteogenic differentiation. Furthermore, our time-course analysis revealed a dynamic in the GS–GL comparison. At baseline (Day 0), chromatin accessibility profiles were nearly indistinguishable between breeds, consistent with the very small number of GS–GL DARs and arguing against strong a priori breed divergence in the resting state. In contrast, GS–GL-associated differences became most apparent at Day 7 following osteogenic induction, when the numbers of DEGs and DARs between GS and GL reached their maximum. These differences then decreased by Days 14 and 21, indicating partial convergence toward a highly similar molecular state as differentiation progressed. We interpret this transient divergence as reflecting variation in early chromatin remodeling responses i.e., differences in responsiveness or remodeling kinetics during early lineage commitment rather than stable baseline differences. This pattern is consistent with reports that MSCs from different donors can exhibit distinct regulatory element activities during early differentiation [75]. Importantly, the magnitude of GS–GL differences remains small relative to the dominant effects of induction state and time. We observed that, the expression of *ALPL*, an early marker of osteogenic differentiation, was lower in GS pigs relative to GL pig, similar with our previous study [9]. Meanwhile, the enrichment of the FoxO and Wnt signaling pathway was revealed at day 7 of induction. FoxOs have been reported to restrain bone formation by the downregulating of Wnt signaling pathway [76]. These variations may underlie the early-stage differences in osteogenic differentiation observed in SMSCs from GS and GL pigs.

## Conclusion

In summary, this study maps the dynamic chromatin accessibility landscape of SMSCs from metabolically distinct donors, revealing epigenetic mechanisms underlying osteogenic differentiation, cell fate decisions, and donor-specific memory. We further identify key regulatory network driving osteogenic differentiation, thereby strengthening the utility of the pig model for bone regeneration research.

## Supplementary Information

Below is the link to the electronic supplementary material.

Supplementary Material 1. Table S1: Summary statistics for RNA-seq and ATAC-seq data. Table S2: Differentially expressed genes (DEGs) in osteogenic-induced vs. control SMSCs at day 7, 14, and 21. Table S3: Differentially expressed genes (DEGs) in GS vs. GL SMSCs at days 0, 7, 14, and 21 of osteogenic induction. Table S4: Top 10 GO and KEGG enriched terms (ranked by fold enrichment) of Differentially expressed genes (DEGs) and differentially accessible regions (DARs) in osteogenic-induced vs. control SMSCs at days 7, 14, and 21. Table S5: GO and KEGG enriched terms of differentially expressed genes (DEGs) and differentially accessible regions (DARs) in GS vs. GL SMSCs at days 7, 14, and 21 of osteogenic induction. Table S6: Differentially accessible regions (DARs) in osteogenic-induced Vs. control SMSCs at days 7, 14, and 21. Table S7: Differentially accessible regions (DARs) in GS vs. GL SMSCs at days 0, 7, 14, and 21 of osteogenic induction. Table S8: Pearson correlations between genes and its corresponding promoter peaks

Supplementary Material 2.Figure S1: Live cell images of control and osteogenic-induced SMSCs isolated from GS and GL pigs at day 0, 7, 14 and 21. (Scale bar: 100 μm). Figure S2. Homer de novo motif enrichment analysis of open and closed DARs identified between GS and GL at each time point after osteogenic induction. Figure S3. Scatter plots showing the overall relationship between DEGs and DARs in the comparisons of breeds according to their log2 fold change. Concordant changes in expression and accessibility are shown in blue and red, whereas discordant changes are shown in purple and orange.

## Data Availability

Raw fastq files and metadata are available in the ArrayExpress database (http://www.ebi.ac.uk/arrayexpress) under accession numbers; E-MTAB-16390 for the RNA-seq data and E-MTAB-16392 for the ATAC-seq data. For reviewer access links: RNA-seq (E-MTAB-16390): [https://www.ebi.ac.uk/biostudies/ArrayExpress/studies/E-MTAB-16390?key=24a89bb1-a2bc-48e8-9ed2-0f29982de024](https:/www.ebi.ac.uk/biostudies/ArrayExpress/studies/E-MTAB-16390?key=24a89bb1-a2bc-48e8-9ed2-0f29982de024)ATAC-seq (E-MTAB-16392): [https://www.ebi.ac.uk/biostudies/ArrayExpress/studies/E-MTAB-16392?key=3b724499-f36d-443f-b560-50bc300d662e](https:/www.ebi.ac.uk/biostudies/ArrayExpress/studies/E-MTAB-16392?key=3b724499-f36d-443f-b560-50bc300d662e).

## Abbreviations

ATAC-seq: Assay for transposase-accessible chromatin using sequencing
DARs: Differentially accessible regions
DEGs: Differentially expressed genes
PCA: Principal component analysis
SMSCs: Synovium derived mesenchymal stem cells
GS: German Saddleback
GL: German Landrace
TFs: Transcription factors
TSS: Transcription start site
Ost: Osteogenic differentiation group
Con: Control group
GRN: Gene regulatory network
ANANSE: ANalysis Algorithm for Networks Specified by Enhancers
