# Supplementary material for: Time-course ATAC-seq and RNA-seq analysis of porcine synovium-derived mesenchymal stem cells under in vitro osteogenic induction
Source: Epigenetics Chromatin. 2026 Apr 1;19:17. doi: 10.1186/s13072-026-00668-z (PMC13085624; doi:10.1186/s13072-026-00668-z)
Supplement: Supplementary file 2 — Supplementary Material 2.Figure S1: Live cell images of control and osteogenic-induced SMSCs isolated from GS and GL pigs at day 0, 7, 14 and 21. (Scale bar: 100 μm). Figure S2. Homer de novo motif enrichment analysis of open and closed DARs identified between GS and GL at each time point after osteogenic induction. Figure S3. Scatter plots showing the overall relationship between DEGs and DARs in the comparisons of breeds according to their log2 fold change. Concordant changes in expression and accessibility are shown in blue and red, whereas discordant changes are shown in purple and orange. [file 13072_2026_668_MOESM2_ESM.docx]

**Time-Course ATAC-seq and RNA-seq Analysis of Porcine Synovium-Derived Mesenchymal Stem Cells from Metabolically Distinct Donors under In Vitro Osteogenic Induction**

Shuaichen Li^1^, Puntita Siengdee^1,2^, Frieder Hadlich^1^, Nares Trakooljul^1^, Michael Oster^1^, Henry Reyer^1^, Klaus Wimmers^1,3^, Siriluck Ponsuksili^1*^

^1^Research Institute for Farm Animal Biology (FBN), Dummerstorf, Germany

^2^Program in Applied Biological Sciences: Environmental Health, Chulabhorn Graduate Institute, Bangkok, Thailand

^3^Faculty of Agriculture, Civil and Environmental Engineering, University of Rostock, Rostock, Germany

**Supplementary Figure S1-3**

**Supplementary Figure S1**


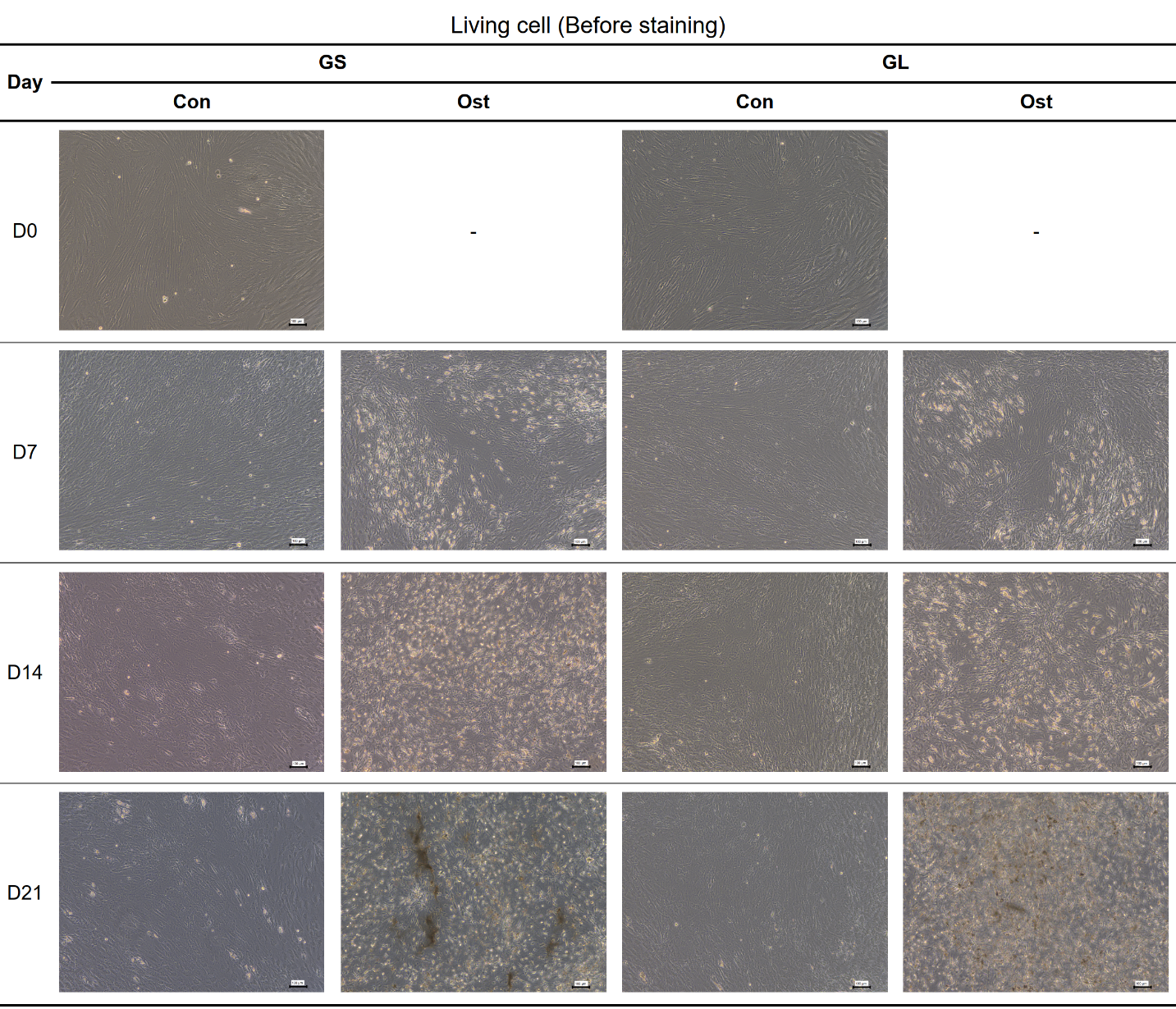


**Supplementary Figure S1.** Live cell images of control and osteogenic-induced SMSCs isolated from GS and GL pigs at day 0, 7, 14 and 21 (Scale bar: 100μm).

**Supplementary Figure S2**


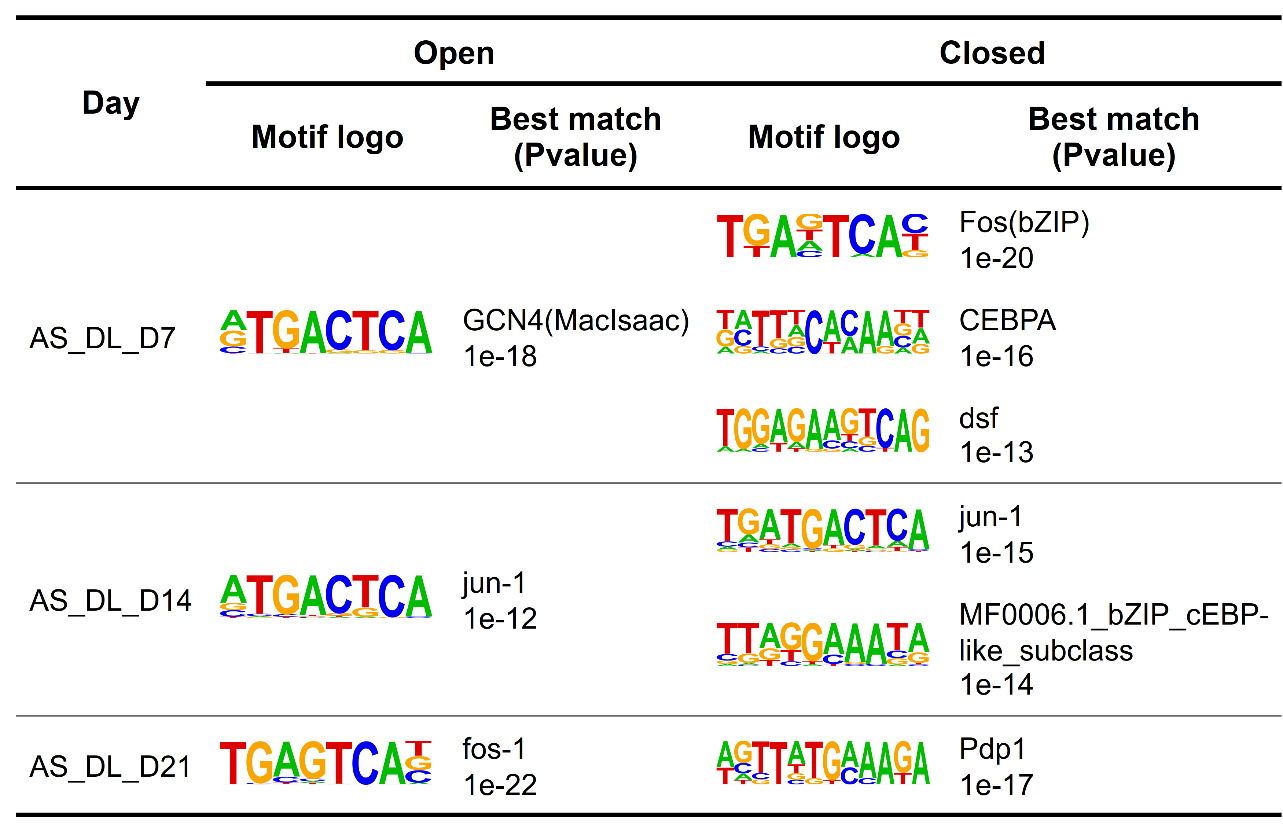


**Supplementary** **Figure S2.** Homer *de novo* motif enrichment analysis of open and closed DARs identified between GS and GL at each time point after osteogenic induction.

**Supplementary Figure S3**


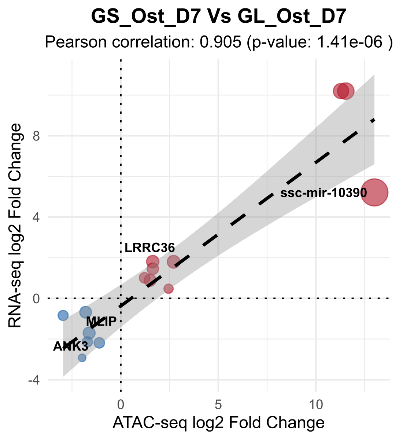

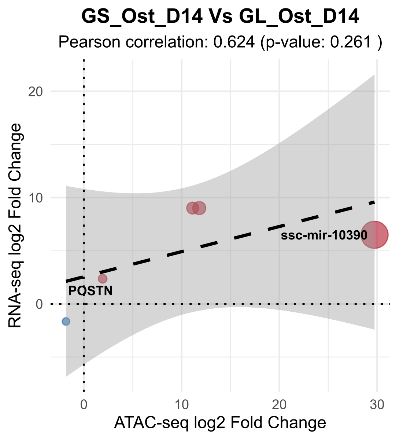

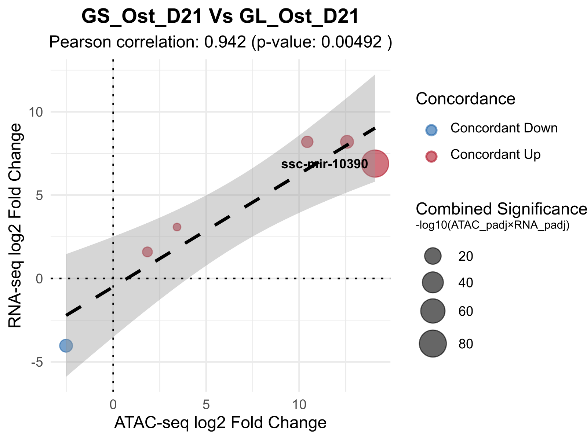


**Supplementary Figure S3.** Scatter plots showing the overall relationship between DEGs and DARs in the comparisons of breeds according to their log2 fold change. Concordant changes in expression and accessibility are shown in blue and red, whereas discordant changes are shown in purple and orange.
